# Validation of the new graded prognostic assessment scale for brain metastases: a multicenter prospective study

**DOI:** 10.1186/1748-717X-6-23

**Published:** 2011-03-02

**Authors:** Salvador Villà, Damien C Weber, Cristina Moretones, Anabel Mañes, Christophe Combescure, Josep Jové, Paloma Puyalto, Patricia Cuadras, Jordi Bruna, Eugènia Verger, Carme Balañà, Francesc Graus

**Affiliations:** 1 Department of Radiation Oncology, Catalan Institute of Oncology, HU Germans Trias, ICO, Badalona, Spain; 2 Department of Radiation Oncology and Clinical Epidemiology, Geneva University Hospital, Geneva, Switzerland; 3 Department of Radiology, HU Germans Trias, ICO, Badalona, Spain; 4 Department of Neurology, HU Bellvitge. L'Hospitalet, Spain; 5 Hospital Clinic, Barcelona, Spain; 6 Department of Neurology, Hospital Clínic, Barcelona, Spain

## Abstract

**Background:**

Prognostic indexes are useful to guide tailored treatment strategies for cancer patients with brain metastasis (BM). We evaluated the new Graded Prognostic Assessment (GPA) scale in a prospective validation study to compare it with two published prognostic indexes.

**Methods:**

A total of 285 newly diagnosed BM (*n *= 85 with synchronous BM) patients, accrued prospectively between 2000 and 2009, were included in this analysis. Mean age was 62 ± 12.0 years. The median KPS and number of BM was 70 (range, 20-100) and 3 (range, 1-50), respectively. The majority of primary tumours were lung (53%), or breast (17%) cancers. Treatment was administered to 255 (89.5%) patients. Only a minority of patients could be classified prospectively in a favourable prognostic class: GPA 3.5-4: 3.9%; recursive partitioning analysis (RPA) 1, 8.4% and Basic Score for BM (BSBM) 3, 9.1%. Mean follow-up (FU) time was 5.2 ± 4.7 months.

**Results:**

During the period of FU, 225 (78.9%) patients died. The 6 months- and 1 year-OS was 36.9% and 17.6%, respectively. On multivariate analysis, performance status (*P *< 0.001), BSBM (*P *< 0.001), Center (*P *= 0.007), RPA (*P *= 0.02) and GPA (*P *= 0.03) were statistically significant for OS. The survival prediction performances' of all indexes were identical. Noteworthy, the significant OS difference observed within 3 months of diagnosis between the BSBM, RPA and GPA classes/groups was not observed after this cut-off time point. Harrell's concordance indexes *C *were 0.58, 0.61 and 0.58 for the GPA, BSBM and RPA, respectively.

**Conclusions:**

Our data suggest that the new GPA index is a valid prognostic index. In this prospective study, the prediction performance was as good as the BSBM or RPA systems. These published indexes may however have limited long term prognostication capability.

## Background

Brain metastasis (BM) is an important and frequent cause of morbidity and mortality in adult cancer patients. The prognosis of BM's patients is usually poor, with a median survival of 1 month and 4 - 6 months in untreated[1] and treated[2] patients, but can be unpredictable in a substantial number of patients[3,4], as a result of patient-heterogeneity within this population. Many clinical factors, not limited to but including performance status, age, extracranial disease and, primary tumour status, have been identified as prognostically relevant. Other factors, such as the number, size or location of BMs, histology of the primary malignancy and interval between primary tumour diagnosis and detection of brain disease have been less considered.

In 1997, the Radiation Therapy Oncology Group (RTOG) published the Recursive Partitioning Analysis (RPA) prognostic index for patients with BMs[5]. It was the first scoring system to classify BM patients in survivorship's categories. The same authors validated this RPA classification 3 years later using results from RTOG 91-04 trial (a randomized study comparing two dose-fractionation schemes) matching with the RPA dataset[6]. This prognostic system was subsequently validated by other authors [7-9]. Based on multivariate analysis of 916 patients, Lutterbach *et al*. suggested the addition of the classification by dividing class III into 3 separate groups was prognostically relevant[4]. Their definition yielded class IIIa defined as age < 65 years, controlled primary tumour and single BM, class IIIc defined as age > 65 years, uncontrolled primary tumour and multiple BM, and class IIIb for all other cases.

In the interim, five new scoring systems have been published since the seminal paper from Gaspar *et al*[5]. In 1999, investigators from Rotterdam proposed a similar score to the RPA[10]. A third parameter (response to steroids before Whole Brain Radiotherapy [WBRT]) was added to performance status (measured by ECOG performance scale) and extent of systemic disease. Two years later, the Score Index for Radiosurgery for BMs (SIR) introduced two new factors, namely the volume and number of BMs[11]. Investigators from Belgium analyzed patients referred to radiosurgery (110 patients with BMs treated with Gamma-knife SRS) in good medical conditions[12]. They did not add new prognostic factors and decided to use a simple score (Basic Score for Brain Metastases [BSBM]), including KPS, extracranial disease (ExCr) and control of primary tumour.

Rades *et al*. developed also a new prognostic index based on 4 parameters[13], three already known (age, KPS, and extracranial metastases) and a new one (i.e. interval from tumour diagnosis to WBRT). These authors replaced primary tumour control by interval from tumour diagnosis to WBRT. This index separated patients into 4 subgroups with significantly different prognosis. The BSBM was recently validated by the same group[14].

Finally, Sperduto *et al.*[15] published an analysis of data from five randomized trials from the RTOG, including RTOG 9508[16]. Their goal was to define the most useful prognostic score by comparing the original RPA[5], the SIR[11], and the BSBM[12] indexes. Importantly, the number of BMs was also considered. Graded Prognostic Assessment (GPA) scores three different values (0, 0.5, or 1). These scores were assigned for each of these 4 parameters: age (> 60, 50-59, < 50), KPS (< 70, 70-80, 90-100), number of BMs (> 3; 2-3; 1), and extracranial metastases (present; not applicable; none). For the authors, the GPA was the most objective, quantitative and easiest to be used. Noteworthy, none of the groups that developed these indexes included all potential prognostic factors in their analysis.

After the publication of Sperduto et al. article[15], we decided prospectively to analyze the GPA index score, compared it to the published BSBM and RPA prognostic indexes and to assess the prediction performances of these three prognostication systems.

## Methods and patients

Two hundred eighty five patients were prospectively entered into this multicentric study investigating the prognostic value of the GPA index[15]. Adult (≥ 18 years) patients were eligible to participate if they had radiologically demonstrable or histologically proven newly-diagnosed BM from a solid tumor. Patients with leptomeningeal carcinomatosis were excluded in this study. Patients were accrued from the Geneva University Hospital (72 patients; 25.3%), and patients from Barcelona area (213 patients; 74.7%): Catalan Institute of Oncology from Badalona (HU Germans Trias; 58 patients), and two prospective GEGB (Barcelona Brain Tumor Group; 155 patients) trials[17].

Investigators scored prospectively BM's patients using the GPA[15], BSBM[12] and RPA[5,6] prognostic indexes and the parameters are detailed in Table 1. The patient's score distributions are detailed in Table 2. Only a minority of patients of our series has been classified in a favourable prognostic class: GPA 3.5-4: 3.9%; RPA 1, 8.4% and BSBM 3, 9.1%.

**Table 1:** Details of the parameters of the RPA [5,6], GPA[15] and BSBM[12] prognostic scales.

Prognostic scale	Parameters	Scores (Class)
**RPA **[5,6]		
	Age < 65 years, KPS ≥ 70, controlled primary tumor, no ExCr	(I)
	All patients not in class I or III	(II)
	KPS < 70	(III)
		
**GPA**[15]		
	≥60/50-59/<50 years (age)	0/0.5/1
	< 70/70-80/90-100 (KPS)	0/0.5/1
	> 3/2-3/1 (# of Brain metastasis)	0/0.5/1
	Present/None (ExCr)	0/1
		
**BSBM**[12]		
	50-70/80-100 (KPS)	0/1
	No/Yes (Controlled of Primary Tumor)	0/1
	Yes/No (ExCr)	0/1

*Abbreviations: *KPS, Karnofsky performance status; BSBM, Basic Score for Brain Metastasis; RPA, Recursive Partitioning Analysis; GPA, Graded Prognostic Assessment; ExCr, Extra-cranial metastatic disease.

**Table 2:** Patient scores distribution

	Number of patients	%
RPA		
1	24	8.4
2	173	60.7
3	88	30.9
		
GPA		
0-1	136	47.7
1.5 - 2.5	124	43.5
3	14	4.9
3.5 - 4	11	3.9
		
BSBM		
0	91	31.9
1	102	35.8
2	66	23.2
3	26	9.1

Total	285	100.0

RPA, Recursive Partitioning Analysis; GPA, Graded Prognostic Assessment; BSBM, Basic Score for Brain Metastases

Median age was 62 years (range, 20 - 90 years) and the median of KPS was 70 (range, 20 - 100). Most patients had primary non small cell lung cancer (43.5%), followed by breast cancer (17.2%), small cell lung cancer (9.5%), colorectal cancer (7.4%) and melanoma (8.6%). Other primary sites were urothelial carcinomas (1.1%), middle gastrointestinal cancers (1.1%), and miscellaneous cancers (11.6%). For the purpose of this analysis, we grouped primary sites as lung (53.0%), breast (17.2%) and others (29.8%).

Date of diagnoses and number of BMs were assessed by neuroimaging. All patients were diagnosed by CT scan (222 patients), MRI scan (211 patients), or both. The median of number of BMs on MRI was 3 (range, 1 - 50). Eighty five patients (29.8%) were diagnosed with synchronous BMs. Forty patients (14.0%) had no ExCr, whereas 115 and 124 had "controlled" disease (40.4%) or progressive disease (43.6%), respectively. Data was not available for 6 (2%) patients. Extracranial metastatic disease (ExCr) was observed in 204 patients (71.6%) and absent in 80 patients (28.1%). For one (0.3%) patient, ExCr status was not available.

Treatment was administered to 255 (89.5%) patients. Table 3 details the Patient's characteristics. As the prognostic indexes were modeled and validated with patients receiving treatment, univariate- and multivariate survival analyses were performed with these individuals (*n *= 255; Table 3). Patients received whole brain radiotherapy (WBRT) with or without boost, radiosurgery (SRS) or involved field radiotherapy, alone or in combination, with or without chemotherapy. Only a few received chemotherapy or surgery alone. The administered treatments are detailed in Table 4. Mean follow-up (FU) time was 5.2 ± 4.7 months. No patient was lost to FU.

**Table 3:** Patient scores distribution

	Number of patients	%
RPA		
1	36	14.6
2	150	60.7
3	61	24.7
Not evaluable	8	3.1
		
GPA		
0-1	103	40.9
1.5 - 2.5	129	51.2
3	13	5.2
3.5 - 4	7	2.8
Not evaluable	3	1.2
		
BSBM		
0	61	24.5
1	80	32.1
2	72	28.9
3	36	14.5
Not evaluable	6	2.4

Total	255	100.0

RPA, Recursive Partitioning Analysis; GPA, Graded Prognostic Assessment; BSBM, Basic Score for Brain Metastases

**Table 4:** Type of treatment

	Number of patients	%
**No treatment**	30	10.5
		
**One Treatment**		
		
WBRT	166	58.2
SRS	8	2.8
S	1	0.4
CT	2	0.7
		
**Combined modality treatment**		
		
*RT combinations*		
WBRT + boost	11	3.9
WBRT + SRS	15	5.3
IFRT + SRS	1	0.4
		
*Postsurgery RT*		
WBRT	2	0.7
SRS	1	0.4
WBRT + SRS	1	0.4
WBRT + boost	6	2.1
		
*RT with CT*		
TMZ + WBRT	40	14
(CDDP + TAX) + WBRT	1	0.4

Total	285	100.0

WBRT, Whole Brain Radiotherapy; SRS, Stereotactic Radiosurgery; S, Surgery; CT, Chemotherapy; RT, Radiotherapy; IFRT, Involved-field exteranl beam RT; TMZ, temozolomide; CDDP, Cisplatin; TAX, Taxans

The purpose of this study was firstly to prospectively validate the GPA prognostic indexes in a multicentric setting. This score was compared to two other published prognostic systems (i.e. BSBM and RPA). Secondly, the prediction performance of these individual indexes was assessed using Harrell's concordance Index *C*[18]. Finally, the time-performance of these indexes were evaluated.

The primary end point for this analysis was overall survival time, calculated from the date of the BM's diagnosis, using the Kaplan-Meier method[19]. The log-rank test, stratified by centers, was used to compare survival distributions and a *P *value < 0.05 was considered statistically significant. Multivariate survival analysis was performed using the Cox proportional hazards model, to calculate hazard ratios (HR) and 95% confidence intervals (CI). The assumption of proportional hazards was checked (test on Schoenfeld residuals[20]). It was not verified for all the prognostic scores, suggesting their prognostic ability changed over time. Thus, the effect of the scores on the survival was modeled by a piecewise constant HR on the time intervals [0-2 months], [2-3 months] and more than 3 months[21,22]. The bounds of the time intervals were selected by a visual inspection of the plots representing the complementary log-log of the survival probabilities *vs*. the logarithm of the time[22]. Factors introduced in the multivariate analyses were prognostic scores, age, number of brain metastases, centers, primary site, tumor control and performance status. To avoid redundancy, when a prognostic score was in the model, the variables involved in this score were excluded from the analysis. The scores introduced in the survival analyses were computed directly from the variables. But the assessment of the scores by clinicians at the time of diagnosis was available and the exact agreement between the defined and re-computed prognostic scores were assessed using the Kappa test[23]. The X^2 ^test was used to compare frequencies between centers, and the Fisher exact test was used when small cell sizes were encountered in 2 × 2 contingency tables. All analyses were performed using the SPSS statistical package (SPSS 17.0, Chicago, IL) and S-Plus 8.0 for Windows (Insightful Corp., Seattle, WA).

## Results

**Figure 1:**
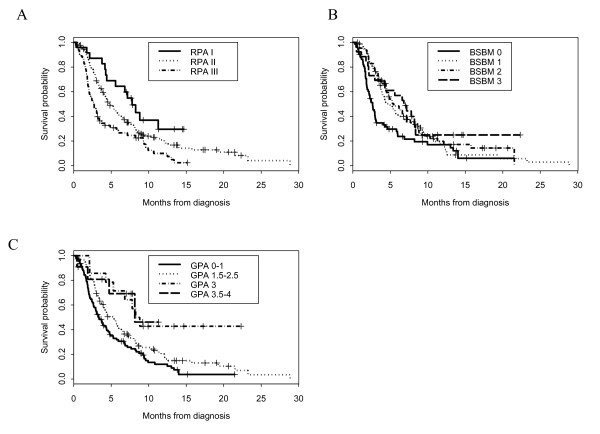
**Actuarial survival curves according to Recursive Partitionning Analysis (A), Basic Score for Brain Metastases (B) and Graded Prognostic assessment (C) class of patients**.

All prognostic indexes were able to predict distinct survival results for BM patients. The overall survival distribution for each prognostic index is shown in Figure 1. The median OS times for the GPA were: Group 0 - 1, 3.3 months; Group 1.5 - 2.5, 5.6 months; Group 3, 7.8 months and Group 3.5 - 4, 8.2 months (Figure 1). Median OS times for the BSBM were: Class 0, 2.6 months; Class 1, 4.4 months; Class 2, 6.8 months and Class 3, 6.8 months (Figure 1). Median OS times for the RPA were: Class 3, 2.5 months; Class 2, 4.8 months and Class 1, 7.2 months (Figure 1). On univariate analysis (OS, log rank test), the worst level of statistical significance between score Groups/Classes was *P *< 0.001 for the GPA, BSBM and RPA indexes, respectively. Other significant identified factors were performance status (*P *< 0.001), center (*P *< 0.001), the presence of ExCr metastasis (*P *= 0.03), control of primary tumor (*P *= 0.04), number of BM (*P *= 0.04). The univariate HRs are shown in Table 5. Age (*P *= 0.97) and synchronous *vs*. metachronous BM (*P *= 0.95) did not reach statistical significance. The results of the multivariate analysis are detailed in Table 5. Factors significantly associated with improved survival were the performance status, center and the three prognostic indexes (Table 5). Primary tumor type was of borderline significance, whereas age and the status of the primary tumor and ExCr disease were not significant. Noteworthy, the performance of the survival prediction was identical among the three prognostic indexes: Harrell's concordance indexes *C *were 0.58, 0.61 and 0.58 for the GPA, BSBM and RPA, respectively.

**Table 5:** Univariate and multivariate analyses for overall survival

	Univariate				Multivariate			
			95% CI				95% CI	
Variable	*P*	HR	Low	High	*P*	HR	Low	High
KPS *								
≤ 60	< 0.001	1			< 0.001	1		
70-80	0.03	0.69	0.50	0.96	0.04	0.70	0.49	0.99
≥ 90	< 0.001	0.35	0.23	0.55	< 0.001	0.40	0.25	0.65

Center *								
1		1				1		
2	< 0.001	0.50	0.35	0.71	0.007	0.59	0.41	0.87

Number of BM *								
> 3	0.04	1			0.59	1		
2 - 3	0.11	0.77	0.55	1.07	0.52	0.89	0.63	1.27
1	0.02	0.64	0.45	0.92	0.33	0.83	0.57	1.21

Primary Tumor *								
Breast	0.05	1			0.08	1		
Lung	0.02	1.59	1.08	2.36	0.04	1.57	1.02	2.41
Other	0.12	1.41	0.92	2.18	0.06	1.55	0.98	2.45

Control Primary *								
NED or SD		1				1		
PD	0.04	1.34	1.01	1.79	0.17	1.24	0.91	1.7

ExCr *								
No		1				1		
Yes	0.03	1.41	1.03	1.92	0.26	1.22	0.86	1.74

Age *								
≥ 60	0.97	1			0.54	1		
50-59	0.81	0.96	0.69	1.34	0.79	0.95	0.67	1.36
< 50	0.92	0.98	0.68	1.41	0.35	1.21	0.81	1.82

RPA **								
I	0.002	1			0.02	1		
II	0.44	1.19	0.77	1.84	0.51	1.16	0.74	1.82
III	0.003	2.08	1.29	3.35	0.01	1.91	0.16	3.14

GPA ***								
3.5 - 4	0.001	1			0.03	1		
3	0.75	1.23	0.33	4.57	0.79	1.19	0.32	4.43
1.5 - 2.5	0.32	1.80	0.57	5.69	0.42	1.62	0.51	5.16
0 - 1	0.07	2.95	0.93	9.34	0.14	2.40	0.74	7.74

BSBM ****								
3	< 0.001	1			< 0.001	1		
2	0.39	0.81	0.50	1.31	0.18	0.71	0.43	1.17
1	0.34	1.25	0.79	1.96	0.70	1.10	0.68	1.78
0	0.002	2.13	1.32	3.44	0.01	1.96	1.17	3.27

*Abbreviations: *KPS, Karnofsky performance status; BSBM, Basic Score for Brain Metastasis; RPA, Recursive Partitioning Analysis; GPA, Graded Prognostic Assessment; BM, Brain Metastasis; ExCr, Extra-cranial metastatic disease.

*: regression model with centers, primary site, its status (control), age, number of brain metastases, KPS and extra-cranial disease

**: RPA was adjusted on centers, primary site and number of brain metastases

***: GPA was adjusted on centers, primary site and its status (control)

****: BSBM was adjusted on centers, primary site, its status (control), age and number of brain metastases

Finally, we performed a Cox-time dependant analysis. All three prognostic indexes best predicted survivorship early as opposed to later in the patient's clinical course. As detailed in Table 6 the significant OS difference observed within 3 months of diagnosis between the various classes/groups among the prognostic scores was not observed after this cut-off time point. The HRs of the GPA (1.5-4.0 *vs*. 0-1), BSBM (≥1 *vs*. 0) and RPA (II *vs*. I) were 1.41 (*P *= 0.1), 1.10 (*P *= 0.76) and 1.13 (*P *= 0.65) after more than 3 months (Table 6).

**Table 6:** Cox time-dependant Multivariate analysis for overall survival

Variable	*P*	HR	Low 95% CI	High 95% CI
GPA *				
1.5-4.0		1		
0-1 [0-2 months]	0.003	2.52	1.38	4.62
0-1 [2-3 months]	0.79	1.09	0.57	2.09
0-1 [> 3 months]	0.10	1.41	0.94	2.11

BSBM **				
≥ 1		1		
0 [0-2 months]	< 0.001	3.62	2.02	6.51
0 [2-3 months]	0.001	2.98	1.53	5.78
0 [> 3 months]	0.76	1.10	0.60	2.02

RPA ***				
I-II		1		
III [0-2 months]	< 0.001	3.27	1.83	5.85
III [2-3 months]	0.22	1.58	0.77	3.24
III [> 3 months]	0.65	1.13	0.67	1.87

*: GPA was adjusted on centers, primary site and its status (control)

**: BSBM was adjusted on centers, primary site, its status (control), age and number of brain metastases

***: RPA was adjusted on centers, primary site and number of brain metastases

The score constructions were problematic in this multicentric prospective study. The prognostics scores were re-computed with the database parameters (i.e. ECrM, control of primary tumor, KPS, Age, number of BMs) and were compared to the score's values attributed by the investigators. Discrepancies (≥ 1 and ≥ 0.5 for the BSBM/RPA and GPA prognostic scores, respectively) were observed in 38 (14.9%), 59 (23.1%) and 25 (9.8%) patients for the GPA, BSBM and RPA prognostic index, respectively. The corresponding κ values were 0.81 (95%CI 0.76-0.87), 0.67 (95%CI 0.60-0.75) and 0.81 (95%CI 0.74-0.88), respectively. Major discrepancies (≥ 2 and ≥ 1.0 for the BSBM/RPA and GPA prognostic scores, respectively) were however rare and observed in only 18 (7.1%), 7 (2.7%) and 0 (0%) patients for the GPA, BSBM and RPA prognostic index, respectively.

## Discussion

In his seminal paper, Sperduto *et al*. compared the newly published GPA with other prognostic indexes, using retrospectively the RTOG database to group BM patients in multiple levels with similar outcome[15]. The authors conclude that the GPA index is as prognostic as the RPA. To our best of our knowledge, this is the first prospective comparison after Nieder's *et al*. retrospective validation[24], of three prognostic indexes in a multinational setting, showing that the GPA, BSBM and RPA are valid tools to prognosticate BM patients.

Our multivariate analysis has shown that three factors, namely, KPS, prognostic scores and center, were significant independent predictors for OS (Table 5). Although the former two parameters were foreseen survivorship predictors, the latter was somewhat unexpected. One center included patients with a significant better KPS (KPS ≥ 70, 81.9% *vs*. 65.3%; *P *= 0.008) and overall prognosis (RPA 3, 18.1% *vs*. 35.2%; *P *= 0.024). Although the goal of prognostication modeling, using a multivariable model, is to provide quantitative knowledge about the probability of outcomes in patients with different characteristics, the present analysis may have been influenced by the recruitment of these patients in this study. One center entered prospectively patients seen routinely in the practice of a busy radiotherapy department, whereas the other Spanish centers entered only a small part of patients in routine clinical practice (*n *= 58 cases). These centers accrued a majority of patients (*n *= 155 cases; 72.8%) in two consecutive prospective trials stemming from the GEGB group. One phase II trial, randomized BM patients to WBRT and temozolomide chemotherapy *vs*. WBRT alone, excluding specifically good prognosis patients who underwent surgery or radiosurgery, with or without RT, and including patients with KPS of 50 to 60[17]. The other trial included patients treated with WBRT to prospectively assess the neurological outcome, excluding specifically patients with good prognosis who underwent surgery or radiosurgery. It is also possible that active palliative care was more readily available in the non-Spanish center, which could have a prognostic impact for these patients[25].

It was assumed that the RPA, when compared to the GPA scoring system, would be more easy to use. We did observe, however, that minor discrepancies (10 - 15%) between the reported and re-computed scores were substantial, irrespective of the three scoring systems. Interestingly, the 4-tier scoring system, i.e. GPA, had the highest number (*n *= 7) of major discrepancies, illustrating the difficulty to evaluate prognosis using a multi level scoring system and non-integer values. Importantly, the measure of agreement between the scored and re-calculated prognostic values was fairly good for the BSBM (κ value of 0.67) and good for the RPA (κ value of 0.81) and GPA (κ value of 0.81), respectively. These observed κ values, assessing the reliability of prospective scoring and retrospective computation for these categorical scales, legitimate the use of these scoring system in daily practice.

Our data suggests that the prediction of these indexes may be for short term (< 2 - 3 months) prognostication only (Table 6). We performed an alternative Cox computation in which the effect of the score on the survival was allowed to vary on time. This approach indicated that the value of the prognostic score at the time of the diagnosis was poorly associated with the survival after 3 months. This observation has not been observed in previous analyses [5,6,10,12], but differential survivorship as a function of classes/groups in these series was assessed using Cox proportional hazards models only. We chose to perform a non-dependant Cox analysis, as the assumption of proportional hazards was not verified in our data, suggesting thus that the classes/groups' prognostic ability changed over time. After visual analysis of the plots, we elected to assess prognostication in three time-intervals, relevant to the clinical outcome of BM patients. This finding was unexpected and should be confirmed by further research in the framework of future prospective trials. It may well be that too few good prognostic patients (in the range of 3% to 15% using the GPA and RPA indexes in our study; Table 3) were included in this analysis with a consequential time-dependence prognostication unreliability of the studied models.

Notwithstanding the data published by Sperduto *et al*. initially in 2008 [15] and updated in 2010 with the incorporation of recent data stemming from prospective randomized trials [26], we cannot state that one prognostication system was superior to another (Figure 1). Small patient numbers and differences in patient populations between these two set of data complicate the interpretation of these findings. The limit of the RPA index have been detailed in a pivotal editorial published by this author[3] and is exemplified by the following clinical case of an asymptomatic young renal cell cancer patient with one brain metastasis, good performance status and two bone metastasis. The predicted survivorship of this very patient would vary more than 4 fold depending on the used prognostic (i.e. RPA or GPA) index. We must be aware however of developing a zealotry about these indexes, in which we, as physician, rely too heavily on them, be it RPA, GPA, BSBM or any other future prognostic scales[3], to tailor our patient's therapy. Median survival of 9.3 years was estimated in 32 BM patients treated in two leading US institutions [27]; among these patients, 9.4% and 28% were older than 65 years and had multiple BMs or systemic disease at brain metastasis, respectively. The majority (60%) of long-term advanced lung cancer patients were older than 65 years in another series[28]. Having said that, there are definite arguments to use a simple prognostic scoring system, using objective (i.e. not using subjective assessment of control of primary tumor) and patient-related parameters to guide the therapeutic management of these challenging patients. More often than not, biases that influence therapeutic decision, made by physicians or family alike, could be diminished by applying selectively prognostic scores to these patients.

In summary, all studied indexes were prognostically relevant in BM patients in this prospective study. Our data did not suggest a greatest prognostic power of one scoring system compared to others. In our study, the significant OS difference observed within 3 months of diagnosis between the BSBM, RPA and GPA classes/groups was however not observed after this cut-off time point. GPA may be more difficult to use for daily prognostication of BM patients. The authors recommend that, regardless of the scoring index used, caution should be exercised by the treating physicians to use discretely these prognostic models and to comprehensively integrate other health, familial and socio-economical related parameters to this very heterogeneous population of patients with BMs.

## Abbreviations

GPA: Graded Prognostic Assessment; BM: brain metastasis; KPS: Karnofsky performance status; ECrM: extracranial metastasis; RPA: recursive partitioning analysis; BSBM: Basic Score for Brain Metastasis; RTOG: Radiation Therapy Oncology Group; WBRT: whole brain radiotherapy; SIR: Score Index for Radiosurgery; GEGB: Barcelona Brain Tumor Group; SRS: stereotactic radiosurgery; HR: Hazard ratio.

## Competing interests

The authors declare that they have no competing interests.

## Authors' contributions

SV and DCW were responsible for the primary concept and the design of the study; SV, DCW, CM, AM, JJ and PP performed the data capture and analysis. SV and DCW drafted the manuscript; DCW and CC performed the statistical analysis; SV and DCW reviewed patient data; all authors revised the manuscript. All authors have read and approved the final manuscript.
